# Fractalkine Expression Induces Endothelial Progenitor Cell Lysis by Natural Killer Cells

**DOI:** 10.1371/journal.pone.0026663

**Published:** 2011-10-24

**Authors:** Dilyana Todorova, Florence Sabatier, Evelyne Doria, Luc Lyonnet, Henri Vacher Coponat, Stéphane Robert, Nicolas Despoix, Tristan Legris, Valérie Moal, Anderson Loundou, Sophie Morange, Yvon Berland, Francoise Dignat George, Stéphane Burtey, Pascale Paul

**Affiliations:** 1 Aix-Marseille University, Laboratoire de Physiopathologie de l'Endothélium –UMR-S 608 INSERM, 13005, Marseille, France; 2 Laboratoire d'Hématologie, CHU Conception, Assistance Publique Hôpitaux de Marseille, Marseille, France; 3 Centre de Néphrologie et de Transplantation rénale, Hôpital de la Conception, Assistance Publique Hôpitaux de Marseille, Marseille, France; 4 Unité d'Aide méthodologique à la Recherche Clinique et Epidémiologique, DRRC, Assistance Publique Hôpitaux de Marseille, Marseille, France; 5 Centre d'Investigation Clinique, Hôpital de la Conception, Marseille, France; Centre de Recherche Public de la Santé (CRP-Santé), Luxembourg

## Abstract

**Background:**

Circulating CD34^+^ cells, a population that includes endothelial progenitors, participate in the maintenance of endothelial integrity. Better understanding of the mechanisms that regulate their survival is crucial to improve their regenerative activity in cardiovascular and renal diseases. Chemokine-receptor cross talk is critical in regulating cell homeostasis. We hypothesized that cell surface expression of the chemokine fractalkine (FKN) could target progenitor cell injury by Natural Killer (NK) cells, thereby limiting their availability for vascular repair.

**Methodology/Principal Findings:**

We show that CD34^+^-derived Endothelial Colony Forming Cells (ECFC) can express FKN in response to TNF-α and IFN-γ inflammatory cytokines and that FKN expression by ECFC stimulates NK cell adhesion, NK cell-mediated ECFC lysis and microparticles release in vitro. The specific involvement of membrane FKN in these processes was demonstrated using FKN-transfected ECFC and anti-FKN blocking antibody. FKN expression was also evidenced on circulating CD34^+^ progenitor cells and was detected at higher frequency in kidney transplant recipients, when compared to healthy controls. The proportion of CD34^+^ cells expressing FKN was identified as an independent variable inversely correlated to CD34^+^ progenitor cell count. We further showed that treatment of CD34^+^ circulating cells isolated from adult blood donors with transplant serum or TNF-α/IFN-γ can induce FKN expression.

**Conclusions:**

Our data highlights a novel mechanism by which FKN expression on CD34^+^ progenitor cells may target their NK cell mediated killing and participate to their immune depletion in transplant recipients. Considering the numerous diseased contexts shown to promote FKN expression, our data identify FKN as a hallmark of altered progenitor cell homeostasis with potential implications in better evaluation of vascular repair in patients.

## Introduction

Endothelium dysfunction plays a central role in the pathogenesis of vascular disorders. During the past decade, evidence has accumulated to show how endothelial integrity results from a critical balance between damage and repair processes. Repair of injured endothelium does not solely depend on vessel wall endothelial cells but also on circulating progenitors originating from the bone marrow or other niches[1]. The CD34^+^ pool of circulating cells encompasses a widely heterogeneous cell population comprising mainly progenitors of hematopoietic lineage paracrinally active in the promotion of endothelial repair, and a scarce fraction of endothelial progenitor cells (EPC) referred as endothelial colony forming cells (ECFC)[2]. EPC have the specific ability to differentiate into endothelial cells and integrate into damaged endothelial layer but also play major role in new vessel formation [3]–[5].

Accumulating evidence indicates that EPC constitute interesting targets for regeneration in cardiovascular and renal disease [6]. Altered EPC numbers and function have been described in patients with renal failure [7]–[9]. This defect could contribute to accelerated arteriosclerosis and high cardiovascular morbidity observed in chronic kidney disease. In the transplantation setting, it was also demonstrated that these progenitor cells have a potential to migrate in human allografts, replace damaged donor endothelium and limit alloimmune injury of graft endothelium [10], [11].

Reduced number and activity of CD34^+^ progenitors are expected to constitute a hallmark of vascular disease progression and transplant damage [12], [13].

Investigation of the role of the chemokine-receptor cross talk in regulating progenitor dependent repair processes has open therapeutic perspectives to favor CD34^+^ regenerative functions as exemplified by studies mainly focused on SDF1-CXCR4 interactions [14], [15].

Fractalkine (FKN) is a membrane-bound chemokine expressed mainly by activated endothelial cells [16], [17] and interacts with its cognate receptor CX3CR1, expressed predominantly on innate immune Natural Killer (NK) cells [18]. FKN induction on mature endothelial cells was shown to enhance NK-cell cytotoxic [19] and proinflammatory cytokine production [20].

The recognition of inhibitory and activating target cell ligands by a wide repertoire of NK cell receptors controls the efficiency of host innate immune responses to neoplastic, infectious and allogeneic challenges [21]. Through sensing of a variety of constitutive or stress-induced ligands on target cells, NK cells act as early cytotoxic effector cells towards recognized targets. While the allogeneic potential of NK cells was mostly investigated in bone marrow transplantation, growing evidence suggests that NK cells may also participate to vascular injury in various pathological settings including atherosclerosis and solid organ rejection [22]–[25].

The FKN/CX3CR1 axis has been involved in the pathogenesis of numerous disorders including atherosclerosis, vasculitis, renal disease and kidney allograft rejection [17], [26]. In addition, CX3CR1 targeted deletion or inhibition of the FKN-CX3CR1 pathway has been reported to prolong survival of cardiac allografts [27], [28].

However, the mechanisms that sustain the role of NK cells in the control of vascular integrity remain poorly addressed and the specific implication of the FKN/CX3CR1 pathway in progenitor cell homeostasis has never been investigated.

We hypothesize that sensing of stress-induced FKN on EPC by innate NK cells could target progenitor cell depletion.

Therefore, we first investigated whether FKN expression can be induced on CD34^+^-derived Endothelial Colony Forming Cells (ECFC) upon inflammatory stimuli. We further evaluated whether up regulation of membrane FKN can activate NK cell adhesion and cytotoxic activity towards progenitor cells *in vitro*. We then investigated whether FKN expression can also be observed on circulating CD34^+^ cells *in vivo* and be associated with lowered progenitor cell count in a cohort of kidney transplant recipients. Finally, we evaluated if serum from transplant recipients and inflammatory cytokines TNF-α and IFN-γ can induce FKN expression on circulating CD34^+^ progenitors isolated from healthy subjects.

## Results

### Inflammatory cytokines induce Fractalkine expression in CD34^+^-derived ECFC

We first investigated whether FKN expression may be induced on CD34^+^-derived endothelial colony forming cells (ECFC) after treatment with inflammatory cytokines TNF-α and IFN-γ.

**Figure 1:**
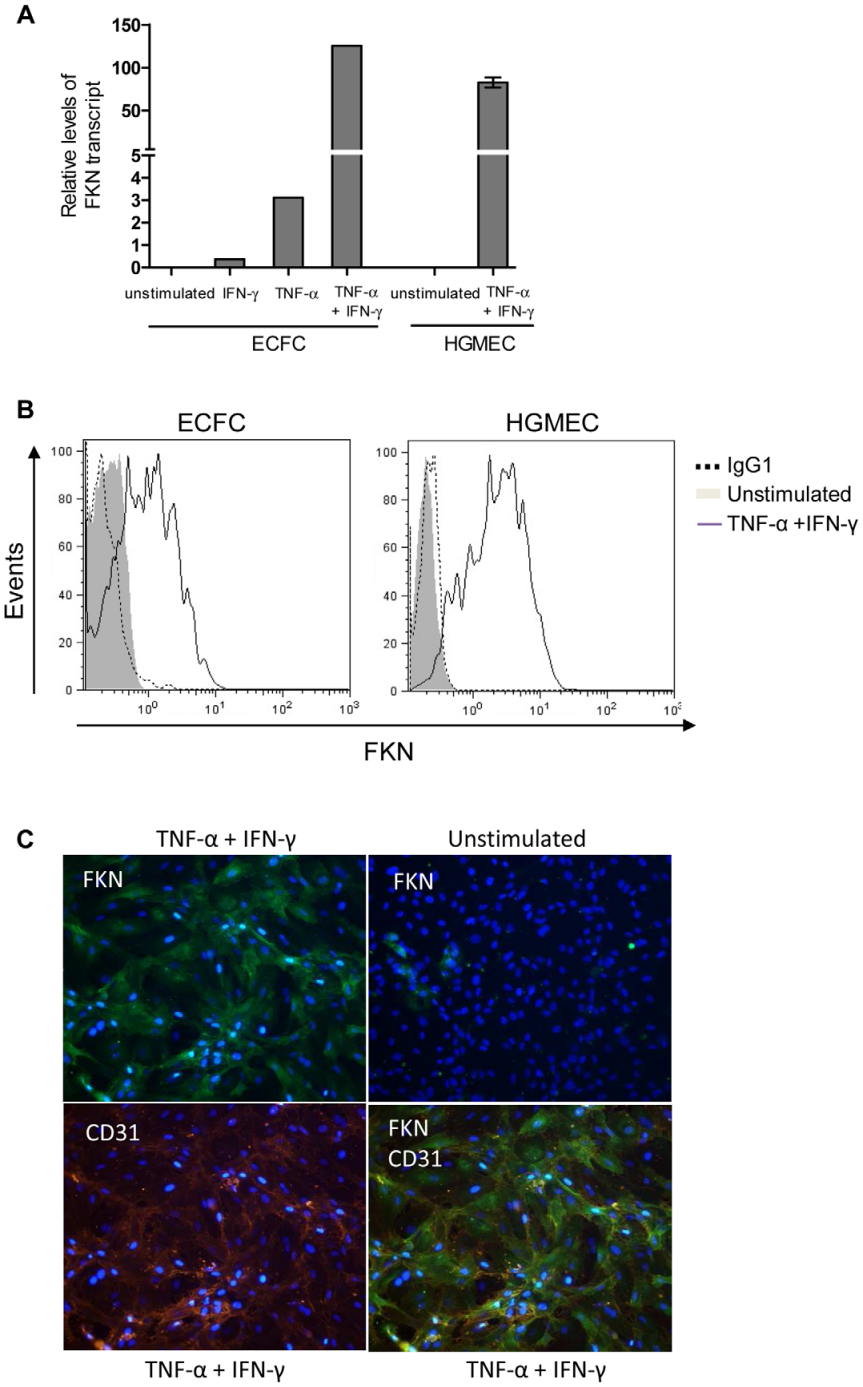
FKN expression is induced in CD34^+^-derived ECFC by inflammatory cytokines. (**A**) Quantitative RT-PCR analysis and (**B**) Representative flow cytometry histograms showing FKN expression in ECFC (n = 6) and HGMEC (n = 5) in response to TNF-α and IFN-γ. (**C**) FKN expression by TNF-α + IFN-γ stimulated ECFC was also assessed by immunofluorescence microscopy, using a goat anti-FKN Ab and revealed by an Alexa 488-conjugated donkey anti-goat Ab. CD31 expression was detected using a mouse anti-CD31 Ab and revealed by an Alexa 546-conjugated goat anti-mouse Ab. The nuclei were stained with Dapi.

While no baseline *FKN* expression was detected in resting endothelial and progenitor cells, TNF-α and IFN-γ induced *FKN* mRNA (Fig. 1A) but also FKN cell surface expression on CD34^+^-derived endothelial colony forming cells, as detected by flow cytometry (Fig. 1B). FKN expression was similarly induced on stimulated glomerular endothelial HGMEC cells used as positive controls. Induction of membrane FKN was consistently observed using distinct batches of ECFC (n = 6) and HGMEC (n = 5). FKN expression on ECFC was confirmed by immunofluorescence microscopy (Fig. 1C), using co-staining with CD31 antibody used to attest the endothelial differentiation of CD34^+^-derived progenitors.

### Fractalkine expression by ECFC enhances NK cell adhesion

**Figure 2:**
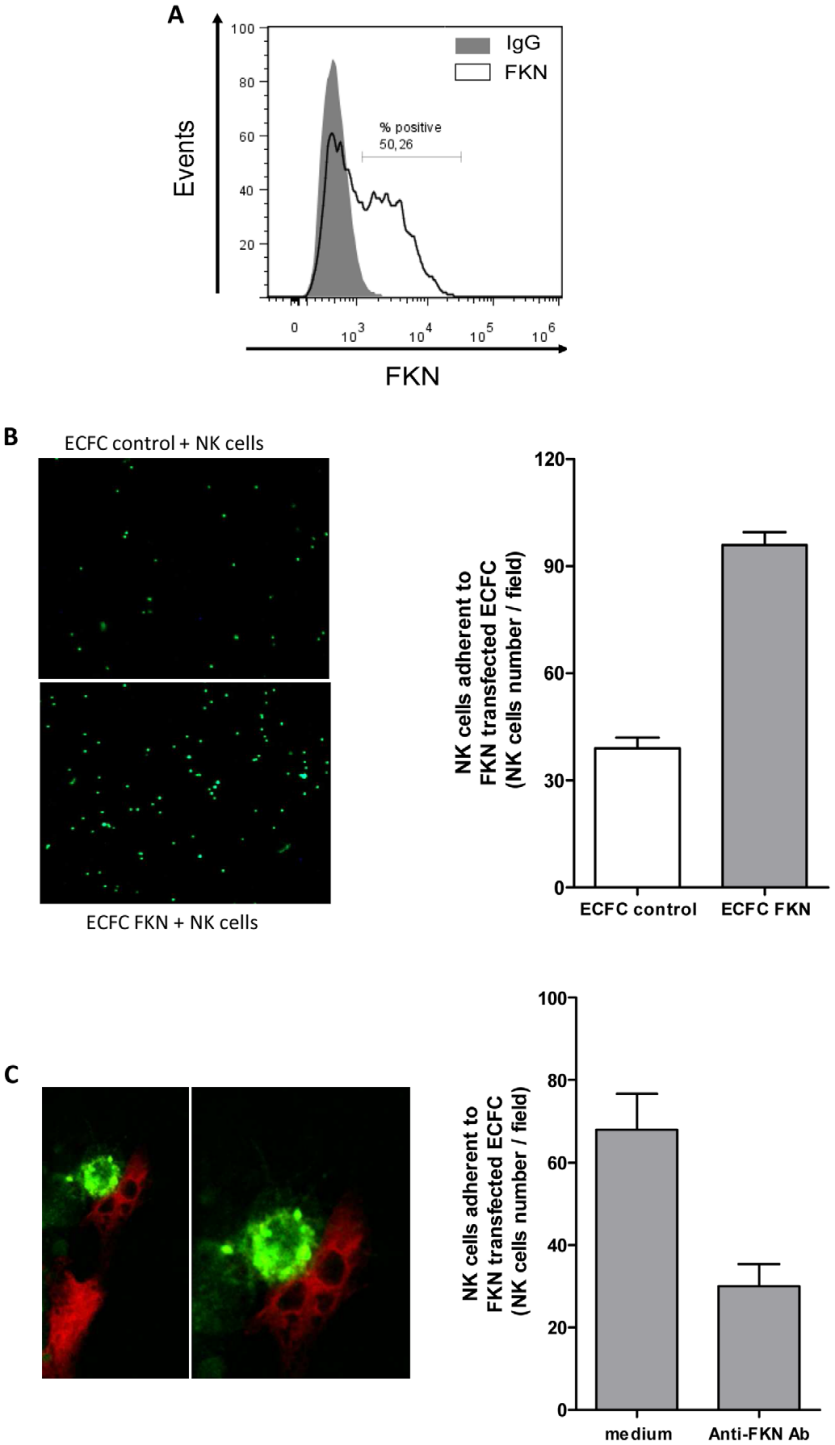
FKN expression by CD34^+^-derived ECFC enhances NK cell adhesion. (**A**) Flow cytometry analysis of FKN expression by ECFC 24 h after transfection with pCDNA3.1 vector encoding membrane FKN. (**B**) FKN- and control empty vector-transfected cells were incubated for 1 h with CMFDA labeled NK cells at an effector: target ratio of 1∶1. (**B, left panel**): NK cell adhesion was analyzed by epi-fluorescence microscopy on an inverted microscope Nikon TE2000-U with a Plan Fluor 4x/0.13 objective. (**B, right panel**): The number of ECFC-adherent NK cells/field was assessed using Image J 1.43 software cell counter. (**C**) Interactions between FKN transfected ECFC and NK cells were detected by confocal microscopy. (**C, left panel**) ECFC transfected with pIRES2-EGFP vector encoding membrane FKN (shown in red) were incubated for 1 h with DiD labelled purified NK cells at an effector: target ratio of 1∶1 (shown in green). (**C, right panel**): FKN-transfected ECFC were treated with 30 µg/ml of anti-human FKN antibody prior incubation with NK cells. The number of ECFC adherent NK cells was assessed by counting DiD positive NK cells/field, using Image J 1.43 software cell counter.

We then investigated the impact of membrane FKN expression in NK cell adhesion to ECFC. As TNF-α/IFN-γ stimulation up-regulated adhesion molecules involved in immune cell adhesion like ICAM-1, VCAM-1, HLA class I (data not shown), the specific involvement of FKN in NK cell adhesion was investigated using membrane FKN-transfected ECFC and purified NK cells. FKN transfection resulted in cell surface expression in 50% of CD34^+^-derived ECFC (Fig. 2A) and led to an increase in NK cell adhesion (Fig. 2B), when compared to adhesion of NK cells to control vector-transfected ECFC. The involvement of FKN in NK cell adhesion to ECFC was further analyzed by confocal microscopy (Fig.2C, left panel). ECFC were transfected with pIRES2 expression vector encoding both membrane FKN and GFP and incubated with purified NK cells labeled with DiD membrane dye. The specific involvement of FKN in NK cell adhesion was confirmed by preincubation of FKN-GFP ECFC with anti-FKN neutralizing antibody that resulted in a 50% reduction of the number of NK cells adherent to FKN-transfected ECFC (Fig. 2C, right panel).

### Fractalkine expression by ECFC enhances their lysis by NK cells

**Figure 3:**
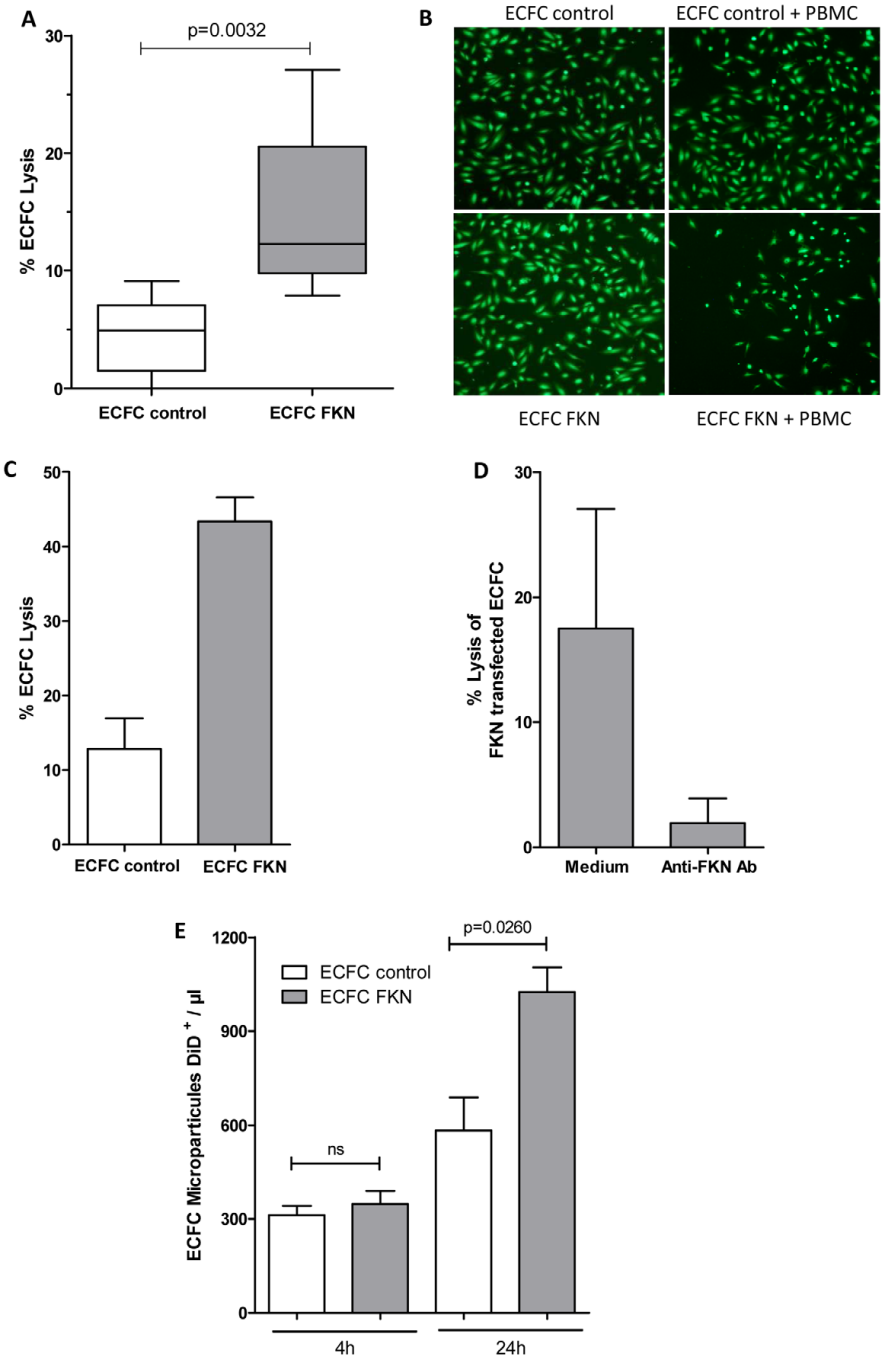
Membrane FKN targets CD34^+^-derived ECFC lysis by NK cells. (**A**) Cytotoxicity assay performed using PBMC effector cells isolated from 7 independent blood donors as described in Methods. Each independent assay was performed in triplicate (n = 7) and analysed using an effector: target ratio = 100∶1. (**B**) Representative illustration of ECFC lysis. CMFDA labeled target cells were analyzed by epi-fluorescence microscopy on an inverted microscope Nikon Eclipse TE 2000-U with a Plan Fluor 4x /0.13 objective, images were acquired using NIS elements AR software. (**C**) FKN enhances ECFC lysis by purified NK cells. CMFDA cytotoxicity assay performed using purified NK cells as effectors, effector: target ratio = 10∶1, (n = 3). (**D**) Addition of FKN neutralizing antibody reduces ECFC lysis. FKN-transfected ECFC were treated with 30 µg/ml of anti-human FKN antibody before addition of PBMC (n = 3). (**E**) Lysis of FKN expressing ECFC resulted in increased microparticles release after 24 h of incubation with PBMC.ECFC were labeled with the lipophilic DiD tracer and transfected with pCDNA3.1 vector coding for membrane FKN (grey bars) or with pCDNA 3.1 empty vector (white bars) and cultured for 24 h. PBMC were added at an effector: target ratio of 50∶1 and co-cultured with target cells for 4 h and 24 h, (n = 5). DiD positive microparticles released by target cells were enumerated by calculation of 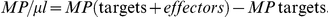

We further investigated whether membrane FKN expression can enhance cytotoxic activity of immune cells towards ECFC and limit progenitor cell survival. We show that membrane FKN transfection renders CD34^+^-derived endothelial progenitors more susceptible to lysis by PBMC effector cells when compared with control vector-transfected ECFC, resulting in a mean 7 fold induction of ECFC cell death (Fig. 3A and 3B). This effect was consistent in independent assays performed in triplicate using PBMC isolated from seven distinct blood donors as effectors and transiently FKN- or control vector- transfected ECFC as targets.

When purified NK cells were added as effector cells, we observed a 4 fold induction of FKN-transfected ECFC lysis when compared to control vector transfected ECFC (Fig. 3C). In addition, preincubation of FKN-transfected ECFC with anti-FKN neutralizing antibody protected ECFC from NK lysis (Fig. 3D). Consistent with FKN-mediated ECFC damage, incubation of FKN-transfected ECFC with PBMC during 24 hours resulted in enhanced microparticles release by FKN-transfected ECFC compared to control vector-transfected ECFC (Fig. 3E).

### The CD34^+^ circulating progenitor cell subset expressing FKN is increased in kidney transplant patients and constitutes an independent factor correlating to lowered CD34^+^ progenitor cell count

**Figure 4:**
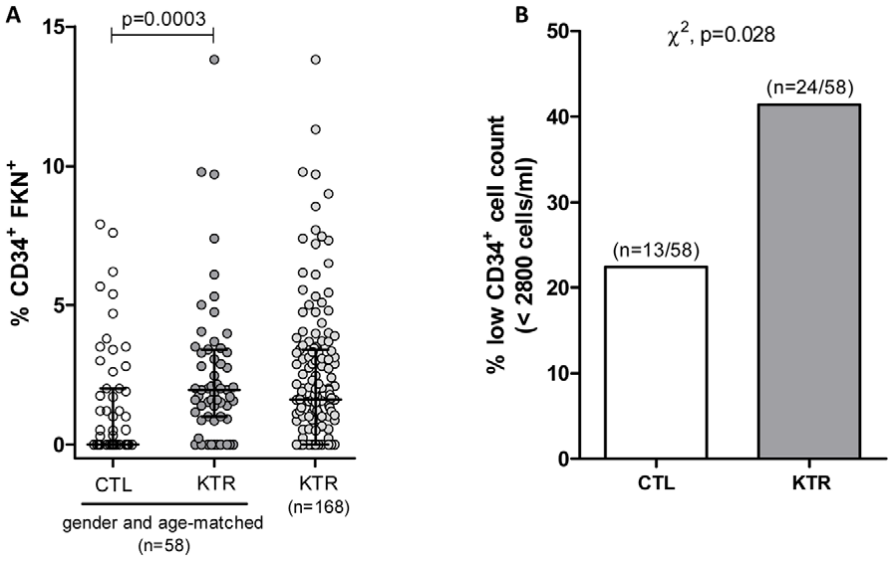
CD34^+^ progenitors expressing FKN are found at higher occurrence in transplant recipients analyzed in reference to gender and matched controls. (**A**) % of CD34^+^ progenitors expressing FKN were evaluated by flow cytometry, in 168 kidney transplant recipients (KTR), and a subgroup of 58 KTR was also analyzed in reference to a gender- and age- matched healthy control group (CTL). (**B**) The % of individuals exhibiting low CD34^+^ cell count is higher in the group of transplant recipients, when analyzed in reference to a group of gender and age-matched controls (n = 58). The 2800 cells/ml cut off value for CD34^+^ cells corresponding to the 25 percentile observed in controls was used as a threshold of low CD34^+^ cell count.

As CD34^+^ circulating progenitor cells are the precursors that give rise to ECFC, we further aimed to determine if FKN expression could also be expressed on circulating CD34^+^ progenitor cells *in vivo*. We therefore conducted a flow cytometry analysis of circulating CD34^+^ and CD34^+^FKN^+^ subsets in a cohort of 168 kidney-transplant recipients (KTR) evaluated at a median of 5.8 years post transplant that allowed detection of FKN expression in a fraction of circulating CD34^+^ cells. The % of CD34^+^ cells expressing FKN (Fig. 4A) and the frequency of CD34^+^FKN^+^ subset was significantly higher in KTR than in gender and age-matched controls (χ^2^, p<0.0001). We then investigated whether FKN expression was associated to a decrease in CD34^+^ progenitors in patients. Individuals with low CD34^+^ counts (threshold cutoff value set at 2800 CD34^+^ cells/ml, the 25 lower percentile of CD34^+^ count observed in controls) were more frequently detected in the KTR cohort (40%) than in the gender and age matched control group (23%; χ^2^, p = 0.028) (Fig. 4B). Consistently, patients with low CD34^+^ levels (<2800 cells/ml) exhibited higher % of FKN expression on CD34^+^ progenitors than KTR with CD34^+^ levels >2800 cells/ml (p = 0.0072).

Multivariate analysis showed that % CD34^+^FKN^+^, age and acute rejection history were independent factors associated with CD34^+^ cell count in transplanted patients (Table 1).

**Table 1:** Univariate and multiple regression analysis explaining CD34^+^ progenitor cells levels analyzed *ex vivo* in Kidney Transplant Recipients.

CD34^+^ cell count	Univariate analysis		Multivariate analysis	
	r Value	Probability	Beta coefficient	Probability
% CD34^+^ FKN^+^	−0.2418	0.002	−0.253	0.001
IS Treatment CSA/Aza	0.0307	0.6972		
Age. yr	−0.1629	0.0395	−0.154	0.046
Gender (female)	−0.0507	0.5241		
BMI	0.189	0.0174	0.204	0.008
Uric acid µmol/l	0.1538	0.0749		
Diabetes antecedents	−0.1425	0.0695		
Acute rejection antecedents	−0.1585	0.0467	−0.157	0.043
			*R2 = 0.122 pANOVA<0.0001*	

Variables that were significantly (p<0.05) associated with CD34^+^ progenitor cell count or marginally significant (*p<*0.20) after univariate analysis were selected in the multivariate model.

### KTR serum and inflammatory cytokines induce FKN expression on CD34^+^ circulating progenitor cells

**Figure 5:**
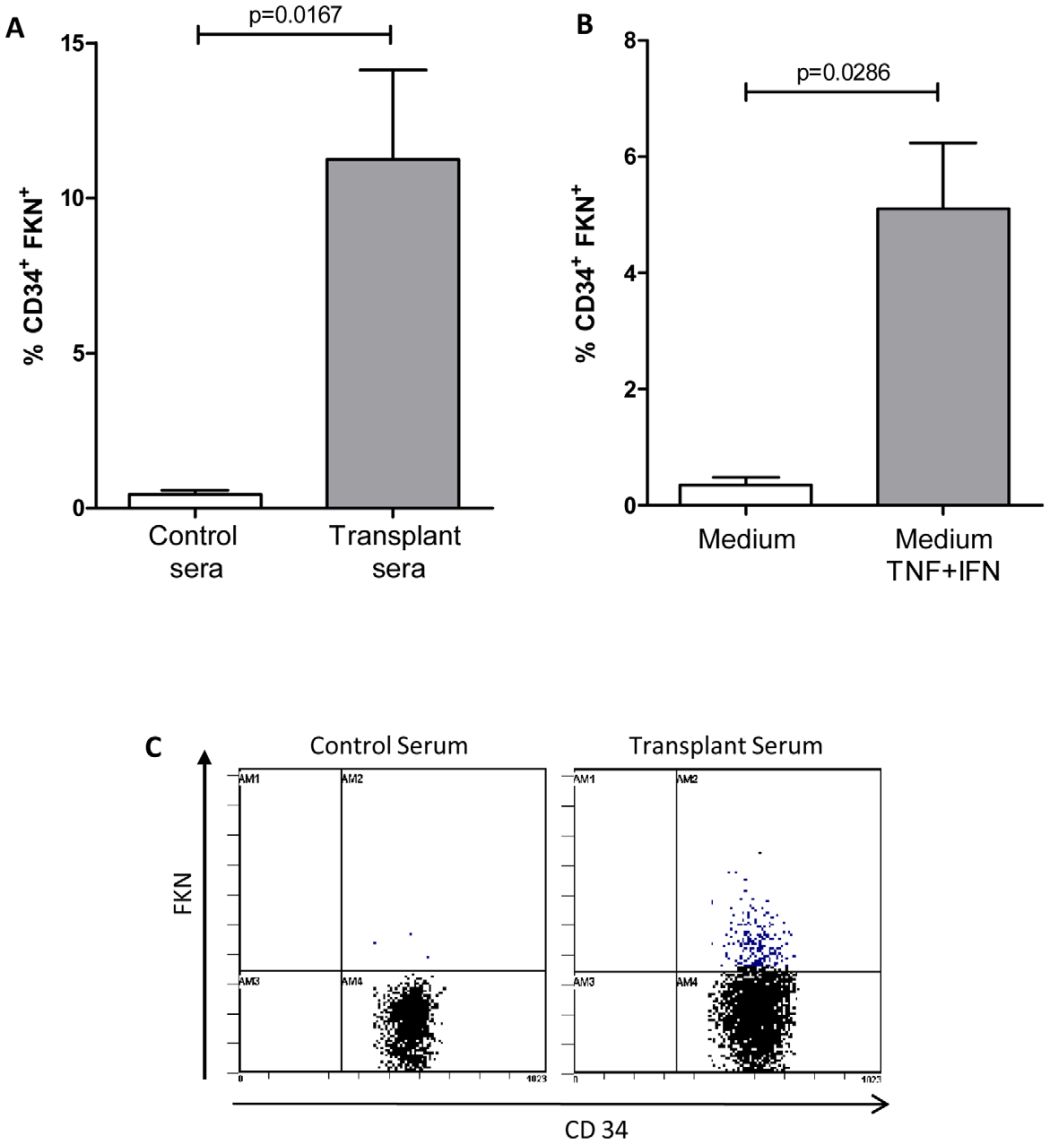
FKN expression is induced on CD34^+^ circulating progenitors by inflammatory cytokines and KTR sera. FKN expression was assessed by flow cytometry after gating of CD34^+^ cells within PBMC isolated from healthy donors after 20 h treatment with (**A**) sera from transplant patients with high % of CD34^+^FKN^+^ cells or with (**B**) TNF-α (20 ng/ml) and IFN-γ (50 ng/ml). Serum from healthy blood donors or control medium served as controls. (**C**) FKN expression was also induced on CD34^+^ cells gated within CD133-purified progenitors after a 20 h treatment with sera from transplant patients.

We further investigated whether soluble factors present in transplant sera from patients with high % of CD34^+^FKN^+^ can induce FKN expression on CD34^+^ progenitor cells freshly isolated from control donors. We show that FKN induction was not restricted to *in vitro* CD34^+^-derived endothelial cell progenitors but was also inducible on CD34^+^ circulating cells. Indeed, flow cytometry analysis showed that serum from KTR (Fig.5A and 5C) but also TNF-α/IFN-γ (Fig. 5B) induced FKN expression on CD34^+^ progenitors gated within PBMC (Fig. 5B) or within CD133^+^ purified progenitor cells (Fig. 5C), while serum collected from CD34^+^ FKN negative healthy donors or control medium did not induce FKN expression (Fig. 5).

## Discussion

Our study unravels a novel mechanism by which Immune NK cells may control endothelial and hematological progenitor cell homeostasis. We show that FKN expression by endothelial progenitor cells leads to their NK mediated-killing in vitro and associates to a reduced number of CD34^+^ cells *in vivo*.

This work is the first to show that membrane fractalkine expression can be induced on both circulating CD34^+^ cells and CD34^+^-derived endothelial progenitor cells. In CD34^+^-derived endothelial progenitor cells with no baseline FKN expression, we show that TNF-α and IFN-γ induce transcriptional and cell surface expression of FKN. We confirmed that such up-regulation was not limited to *in vitro* cultured cells, but could also be observed on purified progenitor cells freshly isolated from healthy adult blood donors upon treatment with serum of KTR patients or TNF-α/IFN-γ.

Previous studies have indeed shown that membrane FKN expression can be induced on mature endothelial cells by proinflammatory cytokines [16], [19]. In line with these data we show that glomerular microvascular endothelial cells, regarded as a major target of immune injury during allograft rejection [29] can also express fractalkine in response to inflammatory stimuli. Through interaction with its cognate receptor CX3CR1, fractalkine expression by endothelial cells was reported to mediate their NK-mediated lysis and also to stimulate interferon-gamma production by innate NK cells [19], [20]. Enhanced FKN and CX3CR1 expression have also been detected in rejected transplant biopsies and associated with cardiovascular disorders, renal inflammation and fibrosis [17], [26], [30]–[32].

Our work thus brings the first evidence that FKN expression is not only a feature of mature cell activation, but may also constitute a signal modulating immune functions toward CD34^+^-derived endothelial progenitors, when these cells are submitted to inflammatory stimuli.

Altogether, these observations led to the hypothesis that FKN up regulation can stimulate cytotoxic NK cell functions towards progenitors and constitute a mechanism affecting their homeostasis.

To sustain this hypothesis, we showed that FKN up regulation on ECFC constitutes a “danger” signal that enhances both NK cell adhesion and cytotoxicity towards ECFC. The specific implication of membrane FKN in targeting NK cell adhesion and NK-mediated ECFC cell death was assessed using FKN-transfected ECFC and anti-FKN blocking antibody. We also provide evidence that FKN mediated ECFC lysis resulted in enhanced ECFC-derived microparticles release. Such microparticles release by apoptotic or activated endothelial cells has been reported as a signature of endothelial injury in various cardiovascular and renal diseases but also as vehicles of coagulation, inflammation, endothelial function and ECFC dependent vascular homeostasis [33], [34]. Altogether, we show that FKN-mediated activation of NK cell cytotoxicity towards ECFC can bypass autologous or allogeneic inhibitory signals delivered to NK cells.

The *in vivo* relevance of FKN expression by CD34^+^ circulating cells and its potential implications in progenitor cell homeostasis was provided in the context of kidney transplant. Detection of CD34^+^ subset expressing high FKN levels was significantly more frequent in transplanted recipients, and rarely observed in healthy blood donors. Moreover, we provide evidence that %CD34^+^FKN^+^ is an independent factor inversely associated with CD34^+^ cell count in a representative cohort of transplant recipients, thus supporting the view that FKN expression may constitute a specific phenotypic feature of CD34^+^ cells in patients with lowered progenitor cell number.

The specific factors that contribute to FKN expression in kidney transplant recipients remain to be characterized. FKN up regulation could be the consequence of inflammation associated with alloimmune conflict and kidney dysfunction.

Indeed serum levels of inflammatory cytokines such as TNF-α were described to be higher in kidney transplant patients and increased significantly during allograft rejection [35]. Sera of patients that exhibit high % of blood CD34^+^FKN^+^ induced FKN expression in circulating CD34^+^ isolated from healthy donors with no baseline FKN expression. This suggests that soluble factors participate to FKN up-regulation in transplanted patients.

Several studies have identified exhaustion of CD34^+^ circulating progenitor cells as a surrogate marker predicting vascular dysfunction, cumulative CV risk and graft vasculopathy [13], [36]–[38]. Definition of mechanisms and markers that characterize the fate of circulating progenitor cells are crucial issues to optimize the prognosis of vascular diseases. There is growing evidence to show that FKN may be involved in atherosclerosis and cardiovascular disease progression [17], [26] but activation of this pathway has never been reported as a mechanism that may impact progenitor cell number. As the FKN-CX3CR1 axis has also been implicated in atherosclerosis progression [17], [39], we expect that FKN-mediated NK cell activation may impair host capacity to counteract onset of atherosclerosis. In the context of kidney transplant, NK cells may thus exert dual deleterious effector function, not only by promoting allogeneic endothelial cell lesion, but also by limiting recipient progenitor cell availability to repair graft and systemic vascular lesions. Monitoring of blood subsets that sign immune tolerance is a challenge to adapt conditioning and immunosuppressive treatment in the clinical setting of hematopoietic and organ transplant. Evaluation of the circulating CD34^+^ compartment may also be of interest in the context of kidney transplant follow-up, notably in novel therapeutic approaches when combined donor hematopoietic stem cell and kidney transplantation are indicated to favor better graft tolerance and immunosuppressive drug withdrawal [40], [41].

Our study highlights the potential value of CD34^+^FKN^+^ as a marker that refines evaluation of the progenitor cell compartment and prompts future prospective studies exploring if evaluation of the circulating CD34^+^FKN^+^ subset can identify patients with lower repair potential and at higher risk to develop CV diseases or renal transplant dysfunction.

In summary, our data support a role for FKN as a gatekeeper controlling immune NK cell activation towards CD34^+^ and endothelial progenitor cells. In response to various pathogenic challenges, NK cells may thus behave as early immune effectors limiting availability of progenitor cells competent for vascular repair. Better understanding of FKN/CX3CR1 dependent mechanisms that render CD34^+^ progenitor cells immunogenic may be of interest to favor stem cell mediated regeneration in various diseases and may contribute to design therapies interfering with the FKN-CX3CR1 pathway to improve graft survival and limit cardiovascular disorders.

## Methods

### Cells and cell culture

Endothelial Colony Forming Cells (ECFC) were generated after density-gradient isolation of human cord blood or peripheral blood mononuclear cells and CD34^+^ cells were purified by magnetic cell separation using the CD34 progenitor-cell isolation kit (Miltenyi Biotech Bergisch-Gladbach, Germany). Cells were plated on 24-wells culture dishes coated with gelatin 0,2 % (Sigma, Saint-Quentin Fallavier) and cultured in endothelial basal medium EBM-2 (Lonza, Saint Bauzire, France) supplemented with 2% fetal bovine serum and an endothelial growth supplement (EGM-2 SingleQuots, Lonza). The medium was replaced every 3 days until the appearance of ECFC colonies. Flow cytometry (flow cytometry) analysis of ECFC showed that these cells express CD34, CXCR4 and endothelial cell markers KDR, VE-cadherin, CD31.

Human glomerular microvascular endothelial cells (HGMEC) were purchased from Cell Systems (Kirkland, WA, USA) and grown according to the manufacturer's instructions.

Purified NK cells were isolated by negative selection using StemSep® Human NK Cell Enrichment Cocktail (StemCell Technologies, Grenoble, France). The purity of CD3^-^ CD56^+^ NK cells was >85% as assessed by flow cytometry.

### Quantitative RT-PCR analysis

Total RNA was extracted from ECFC and HGMEC using the RNA extraction kit (Qiagen, Courtaboeuf, France) and 3 µg of RNA was reverse-transcribed to cDNA according to the manufacturer's instructions, using random hexamers (Invitrogen). cDNA was analyzed for expression of *FKN/CX3CL1* transcript by real time PCR using FastStart DNA Master ^PLUS^SybrGreen I reaction Mix on a Light Cycler® 480 (Roche Applied Science, Meylan, France). The expression of *FKN* transcript was normalized to the expression of the housekeeping gene *HPRT*. The sequences of oligonucleotide primers used for *CX3CL1* and *HPRT* amplification were: *CX3CL1*forward: 5′-ACGAAATGCAACATCACGTGC-3′; *CX3CL1*reverse: 5′-TCCAAGATGATTGCGCGTT-3′; *HPRT*forward: 5′-GAGCTATTGTAATGACCAGTCAACAGGG-3′; *HPRT*reverse: 5′- GGATTATACTGCCTGACCAAGGAAAGC-3′. The annealing reactions were carried out at 65°C.

### Immunofluorescence microscopy

Cord blood-derived ECFC were grown in gelatin 0.2% (Sigma- Aldrich) coated Culture Slides BD Falcon™ until they reached confluence and stimulated with IFN-γ (50 ng/ml) and TNF-α (20 ng/ml) for 16 h, unstimulated ECFC serve as control. Cells were fixed with 1% paraformaldehyde (PFA) solution for 10 min. To prevent unspecific binding, ECFC were incubated for 1 h at room temperature with PBS containing 3% BSA and 1% SVF. Then cells were incubated with a goat anti-human CX3CL1 Ab, 5 µg/mL (R&D Systems) for 30 min at 4°C, followed by an Alexa 488-conjugated donkey anti-goat Ab, 2 µg/mL (Molecular Probes, Invitrogen). Cells were fixed with 1% PFA for 10 min and further incubated with a mouse anti-human CD31 Ab, 5 µg/mL (Immunotech, Beckman Coulter), followed by an Alexa 546-coupled goat anti-mouse Ab, 2 µg/mL (Molecular Probes, Invitrogen). Furthermore, DAPI staining of cell nuclei was performed for 10 min at room temperature. Samples were analyzed by epi-fluorescence microscopy on an inverted microscope Nikon Eclipse TE2000-U with a Plan Fluor 20x/0.45 objective, images were acquired using NIS elements AR software.

### Expression plasmid constructs and ECFC transfection

The DNA fragment encoding membrane FKN was amplified from FKN cDNA by PCR using following primers: 5′*NheI*-CX3CL1 primer (5′-GCTAGCCAGCCATGGCTCCGATATCTC-3′); 3′CX3CL1-*NotI* primer (5′-GCGGCCGCTCACACGGGCACCAGGA-3′). FKN cDNA was cloned into mammalian expression vector pCDNA3.1 (-) Hygro (Invitrogen, CergyPontoise, France) using NheI-NotI restriction sites and into pIRES_2_EGFP vector (Clontech, Ca, USA) using NheI-BamHI restriction sites. After amplification in *E.coli* strain Top 10 (Invitrogen), plasmid DNA was purified using Endofree-Plasmid-Maxi-Preparation kit (Qiagen, Courtaboeuf, France) according to the manufacturer's instructions. For transfection, ECFC were suspended in nucleofector kit OLD solution (Lonza, Saint Bauzire, France), mixed with 2 µg of pCDNA3.1 empty vector or pCDNA 3.1 coding for membrane FKN and transfected using Amaxa Nucleofector apparatus II (Lonza) and U-001 pulsing parameters. FKN expression was evaluated by flow cytometry 24 h after nucleofection.

### NK cell Adhesion assay

24 h prior to adhesion assay, cord blood-derived ECFC were transfected with pCDNA3.1 (-) empty vector or with pCDNA3.1 (-) encoding membrane FKN and plated onto 96-well plate at 10×10^3^ cells/well in EGM-2 medium and incubated for 24 h at 37°C in 5% CO_2_. Transfected cells were incubated for 1 h with CMFDA labeled purified NK cells at 1∶1 ratio. NK cell adhesion was analyzed by epi-fluorescence microscopy on an inverted microscope Nikon TE2000-U with a Plan Fluor 4x/0.13 objective. The number of ECFC-adherent NK cells/field was assessed using Image J 1.4 software cell counter. To assay the specific role of FKN in ECFC/NK cell interactions, adhesion assays were also performed using ECFC transfected with pIRES2-EGFP vector coding for both GFP and FKN and transfected ECFC were incubated with 30 µg/ml of FKN neutralizing antibody (R&D Systems) for 30 minutes at 37°C, 5% CO_2_. Then purified NK cells stained with 8 µg/ml DiD lipophilic tracer were incubated with FKN transfected ECFC for 1 h at effector: target ratio of 1∶1. The number of ECFC-adherent NK cells/field was assessed and ECFC-NK cell interactions were imaged by resonance laser scanning confocal microscopy (SP5 Leica) using excitation wavelengths of 488 and 633 nm with 63x oil immersion objective (N.A. = 1.4).

### Evaluation of ECFC lysis

CD34^+^-derived ECFC were transfected with pCDNA3.1(-) empty vector or with pCDNA3.1(-) coding for membrane FKN, plated onto 96-well plate at 10×10^3^ cells/well in EGM-2 medium and incubated for 24 h at 37°C in 5% CO_2_. Target cells (transfected ECFC) were labeled with 5 µM CellTracker™ Green CMFDA (Invitrogen) and incubated with PBMC at effector: target ratio of 50∶1 for 4 h at 37°C in 5% CO_2_. After removing of medium, the fluorescence of remaining adherent ECFC was measured using PerSeptiveBiosystems Cytofluor4000 multi-plate cell reader (Applied Biosystems, Courtaboeuf, France). Assays were performed in triplicate, and data were expressed as % of ECFC lysis:
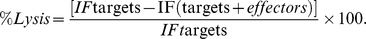



Each well was also analyzed by epi-fluorescence microscopy on an inverted microscope Nikon Eclipse TE2000-U with a Plan Fluor 4x /0.13 objective, images were acquired using NIS elements AR software.

### Microparticle release assay

24 h prior to test, CD34^+^-derived ECFC were labeled with Lipophilic Tracer DiD (Molecular Probes, Invitrogen) according to the manufacturer's instructions. Cells were transfected with pCDNA3.1 empty vector or with pCDNA3.1 encoding membrane FKN, seeded onto 96-well and cultured in EGM-2 medium for 24 h at 37°C in 5% CO_2_. PBMC effector cells were added at effector: target ratio of 50∶1 and incubated with target cells for 4 h and 24 h in RPMI medium containing 5% SVF. After incubation, culture medium was harvested, centrifuged 2×5 minutes at 300 g and 10 minutes at 1500 g to discard PBMC and cell debris and supernatants were collected for flow cytometry analysis of ECFC-derived microparticles. Using 0.5 and 1.0 µm latex beads as gating parameters, EMP were defined as particles ≤1 µm size and enumerated from the region corresponding to DiD^+^/Annexin^+^ events on a Gallios cytometer (Beckman Coulter) after adding 30 µL of counting beads at concentration of 960 beads/µL (FlowCount™Fluorospheres, BeckmanCoulter) to 30 µL of each sample. Microparticles count was expressed as absolute numbers per microliter of supernatant.

### Flow cytometry analysis of FKN induction on CD34^+^ circulating and ECFC progenitor cells

Membrane FKN expression was analyzed by flow cytometry after gating on CD34^+^ 7AAD^-^ viable circulating progenitors within PBMC or CD133-purified cells. Briefly, CD133**^+^** cells were purified from peripheral blood of healthy donors by magnetic cell separation using the CD133 progenitor-cell isolation kit (Miltenyi Biotech Bergisch-Gladbach, Germany). FKN induction was analyzed after 20 h stimulation of CD133^+^ cells or PBMC with StemSpan medium (Stem Cell Technologies), medium containing TNF-α (20 ng/ml) and IFN-γ (50 ng/ml) or 50% serum from transplant patients or healthy blood donors. CD34^+^-derived endothelial colony forming cells (ECFC) were isolated from umbilical cord blood from healthy volunteers and cultured as previously described [42]. For FKN induction, cells at passage 2 to 4 were starved for 4 h in EBM-2 supplemented with 1% BSA and then stimulated with IFN-γ (50 ng/ml), TNF-α (20 ng/ml) or with a combination of IFN-γ+TNF-α for 16 h (mRNA analysis) or for 24 h (protein analysis). After stimulation cells were trypsinized and stained with a mouse anti-human FKN-PE or with mouse IgG1-PE (R&D Systems) and analyzed on a FC500 cytometer (Beckman Coulter).

### Patients and Ethics Statement

All patients and healthy volunteers who participated in this study signed an informed consent. The clinical research protocol supervised by Institut National de la Santé et de la Recherche Médicale (Granted in 2008 under ref ID RCB 2008-A00604-51, C07-17) has obtained approval from the following ethics committee: Agence Française de sécurité sanitaire (Afssaps Ref B805-1860) and Comité de Protection des Personnes Sud Méditerranée I, Marseille France (CPP Sud Méditerranée) to insure that procedures were conducted in strict accordance with the Declaration of Helsinki principles. From November 2008 to November 2010, 168 kidney transplant recipients (KTR) followed in the transplant unit of Centre de Nephrology et Transplantation Rénale, Hôpital de la Conception Marseilles, France were prospectively included in this study after written informed consent. Median time post transplant was 5.8 years post transplant (25–75 percentile range: 4.6–7.1). Alternative immunosuppressive regimen used since transplant was either Cyclosporine +Azathioprine (CSA/Aza, 53.6%) or Tacrolimus + MycophenolateMofetil (TAC/MMF, 46.4). Median BMI of transplanted patients was 25 (25–75 percentile range: 22.4–27.2). Median recipient age was 54.2 (25–75 percentile range: 47–61) and the analyzed cohort consisted of 107 male and 61 females. Estimated Glomerular filtration rate (eGFR) was calculated using the simplified MDRD equation (mean eGFR: 53.5, SD: 21.9). Graft rejection episodes since transplant were detected in 11% of patients for acute rejection and 23.4 % for chronic rejection. A control group was composed of age- and gender-matched healthy volunteer blood donors (n = 58) without signs of renal failure. The control group was composed of 31 men and 27 women, mean age 50.6 yrs (SD = 9.16) and eGFR: 98.9 ml/mn/1.73 m^2^mean (SD = 17.15).

### Flow cytometry (flow cytometry) analysis of CD34^+^ cell count and FKN expression on CD34^+^ cells in KTR

CD34^+^ progenitors were analyzed within peripheral blood mononuclear cells (PBMC) isolated from KTR and healthy donors after density gradient centrifugation using lymphocyte separation medium (Eurobio, Courtaboeuf, France). CD34^+^ cells were enumerated using Stem-Kit™ (Beckman Coulter) as described [8]. PBMC were stained with CD34-FITC, 7-AAD viability marker (Beckman Coulter, Marseille, France) and FKN-PE antibodies (R&D Systems, Minneapolis, MN, USA). Expression of FKN (CX3CL1) was analyzed on a FC500 cytometer (Beckman Coulter) using a two-dimensional side scatter-fluorescence dot plot after gating on 7-AAD negative viable CD34^+^ cells. Flow cytometry analysis of NK cells within PBMC was performed as described [43].

### Statistical analysis

Statistical analysis was performed using PASW Statistics version 17.0.2 (SPSS Inc., Chicago, IL, USA). Continuous variables were tested for normal distribution with the Shapiro-Wilk normality test and were expressed as mean ± SD or as median with interquartile range according to their distribution. Categorical variables were reported as count or percentages. Association between continuous variables was analyzed using Spearman's Rank Correlation test. The Pearson Chi-square test was used to check whether two categorical variables are statistically associated. For continuous variables, the medians for two groups were compared using non-parametric Mann-Whitney test. The variables found to significantly associate (*p<*0.05) with outcomes variables or marginally significant (*p<*0.20) in the univariate analysis, or that had a clinical relevance were selected in the multivariate model. Backward multiple regression was used to construct models associating CD34^+^ cell count and %FKN^+^CD34^+^ with explanatory variables.
